# PLK1 overexpression as a dual-role biomarker and therapeutic vulnerability in pulmonary adenocarcinoma

**DOI:** 10.7717/peerj.20618

**Published:** 2026-01-15

**Authors:** Lukuan You, Yinmei Xu, Yankan Fu, Jianxiong Li

**Affiliations:** 1 Senior Department of Oncology, Chinese PLA General Hospital, Beijing, China; 2 Chinese PLA General Hospital (PLA Medical School), Beijing, China

**Keywords:** PLK1, LUAD, Prognosis, Immune infiltration, Cell cycle

## Abstract

**Background:**

PLK1 is associated with various malignant tumors, but its correlation with lung adenocarcinoma (LUAD) remains unclear. This research seeks to explore the differences in PLK1 expression in LUAD and evaluate the relationship between PLK1 expression and the outcomes for LUAD patients.

**Methods:**

Information on LUAD patients was sourced from The Cancer Genome Atlas (TCGA), Gene Expression Omnibus (GEO), and Genotype-Tissue Expression (GTEx). The XianTao Academic Online Platform was employed for systematic analysis of PLK1, including: (1) Wilcoxon rank-sum test to compare PLK1 expression between LUAD and normal tissues; (2) logistic regression analysis evaluating PLK1-clinicopathological feature relationships; (3) Kaplan–Meier and COX regression analyses assessing prognostic significance; (4) nomogram construction for survival prediction. Immunohistochemical (IHC) staining results from the Human Protein Atlas (HPA) validated PLK1 protein expression. Functional characterization using the XianTao platform included: (1) Analysis of PLK1-coexpressed genes using Gene Ontology (GO) and Kyoto Encyclopedia of Genes and Genomes (KEGG) pathways; (2) single-sample gene set enrichment analysis (ssGSEA) to measure immune cell infiltration in tumors with high PLK1 expression. *In vitro* validation included: (1) cell proliferation assessment (CCK-8 and colony formation assays); (2) apoptosis detection *via* Annexin V/PI staining; (3) cell cycle analysis by PI staining flow cytometry; (4) cell cycle-related protein expression evaluation using Western blotting (Cyclin B1, CDK1).

**Results:**

PLK1 expression was significantly elevated in LUAD tumor tissues compared to adjacent normal samples across multiple cohorts. Elevated PLK1 expression was strongly associated with advanced clinicopathological stages (tumor/node/metastasis (T/N/M)) and poorer overall survival. Functional enrichment analysis revealed that genes co-expressed with PLK1 were predominantly involved in cell cycle regulatory pathways. Furthermore, transcriptomic profiling indicated a significant correlation between high PLK1 expression and an immunosuppressive tumor microenvironment. Experimental validation in A549 cells demonstrated that pharmacological inhibition of PLK1 (*via* GSK461364) effectively suppressed cell proliferation, induced G2/M phase arrest, promoted apoptosis, and led to the accumulation of Cyclin B1 and CDK1 proteins.

**Conclusion:**

PLK1 overexpression signifies aggressive disease and poor prognosis in LUAD, mechanistically linked to cell cycle dysregulation and an immunosuppressive microenvironment. Our findings nominate PLK1 as a promising therapeutic target and biomarker, warranting further investigation into PLK1-directed therapies.

## Introduction

Lung adenocarcinoma (LUAD), comprising 40–50% of non-small cell carcinoma (NSCLC) subtypes, constitutes a major contributor to NSCLC-related mortality, with this disease entity accounting for >1.8 million global fatalities annually (Molina et al., 2008; Bray et al., 2024). While targeted therapies against driver mutations (*e.g.*, epidermal growth factor receptor/anaplastic lymphoma kinase (EGFR/ALK)/ALK) and immune checkpoint inhibitors improve outcome, resistance remains elusive. About 30% of EGFR-mutant LUAD patients are primary resistant to first-generation tyrosine kinase inhibitors (TKIs), and almost all responders develop resistance *via* T790M mutation (Chen & Riess, 2020; Laface et al., 2023). PD-1/PD-L1 inhibitors benefit only 20%–30% of LUAD patients (Tsao et al., 2017; Song et al., 2019), highlighting the need for novel therapeutic targets.

Polo-like kinase 1 (PLK1), a master regulator of mitotic progression, orchestrates G2/M transition, centrosome maturation, and chromosome segregation through phosphorylation of key substrates (*e.g.*, CDC25C, Cyclin B1, BUBR1), and its overexpression in solid tumors is strongly associated with genomic instability, chemoresistance, and poor prognosis (Seki et al., 2008; Beck et al., 2013; McGourty & Rape, 2013). Beyond its canonical cell cycle roles, emerging evidence reveals tissue-specific oncogenic mechanisms of PLK1. In breast cancer, PLK1-mediated BRCA1 phosphorylation impairs DNA repair, sensitizing tumors to PARP inhibitors (Siraj et al., 2023). Similarly, in prostate cancer, PLK1 activates the MEK/ERK/Fra1-ZEB1/2 axis to drive epithelial-mesenchymal transition (EMT) and metastasis (Wu et al., 2016).

The compelling oncogenic functions of PLK1 across cancer types have spurred the development of targeted inhibitors to explore its therapeutic potential. Notably, recent preclinical evidence has begun to elucidate the direct anti-tumor activity of these agents in LUAD. For instance, onvansertib, a highly selective PLK1 inhibitor, has been demonstrated to significantly suppress the proliferation and migration of LUAD cells, induce G2/M phase arrest and apoptosis, and overcome cisplatin resistance by regulating the β-catenin/c-Myc signaling pathway. Its efficacy has been confirmed in both xenograft and patient-derived xenograft (PDX) models, providing a strong rationale for targeting PLK1 in this malignancy (Wang et al., 2023).

Translating these findings clinically, a randomized phase II trial (Ellis et al., 2015) evaluated volasertib—another potent and selective PLK1 inhibitor—in combination with pemetrexed *versus* pemetrexed monotherapy as a second-line treatment for advanced NSCLC. Although the combination did not significantly improve progression-free survival (median 3.3 *vs.* 5.3 months), it demonstrated a notably higher objective response rate (21.3% *vs.* 10.6%). This suggests a biologically relevant antitumor effect in a subset of patients (Ellis et al., 2015), underscoring the promise of PLK1 inhibition while simultaneously highlighting the challenge of tumor heterogeneity and the need for predictive biomarkers.

This heterogeneity is reflected in the complex, context-dependent functional role of PLK1 in LUAD, which is influenced by genetic background (*e.g.*, KRAS/p53 *versus* EGFR mutations) and components of the tumor microenvironment (TME). For instance, KRAS/p53 co-mutant subtypes rely on PLK1-driven oncogenesis, whereas EGFR-mutant tumors show paradoxical responses, potentially mediated by PLK1-EGFR crosstalk (Kong et al., 2022). PLK1 is suggested to have dual immunomodulatory effects: its activity may promote an immunosuppressive stroma (*e.g.*, by potentially facilitating M2 macrophage polarization *via* CXCL2 secretion), while its inhibition has been reported to trigger immunogenic cell death (ICD), thereby enhancing T-cell infiltration (Zhou et al., 2021).

Furthermore, PLK1 plays a pivotal role in mediating radioresistance, a major clinical challenge in LUAD management. Its activity is required for DNA damage checkpoint recovery following genotoxic stress, such as that induced by ionizing radiation (Wakida et al., 2017). Inhibition of PLK1 by agents like volasertib abrogates this recovery mechanism, potentially locking cells in a radiosensitive G2/M phase and enhancing mitotic catastrophe and apoptosis (Dong et al., 2018). Preclinical studies in squamous cell carcinoma models have demonstrated that the application of volasertib during fractionated irradiation significantly reduced tumor growth compared to irradiation alone (Lund-Andersen et al., 2014), providing a strong rationale for combining PLK1 inhibition with radiotherapy to overcome radioresistance.

Emerging evidence suggests non-canonical PLK1 functions, such as PD-L1 upregulation *via* NF-κB pathway activation (Zhang et al., 2022a; Zhang et al., 2022b) and spatial modulation of cytotoxic T-cell exclusion through fibroblast interactions (Barbosa Rabago et al., 2021). However, these mechanisms remain underexplored in LUAD progression, highlighting four critical gaps: (1) limited systems-level analysis of PLK1-associated transcriptional networks; (2) insufficient understanding of PLK1-TME crosstalk, particularly its role in immunosuppressive cell recruitment; (3) limited functional validation in preclinical models reflecting LUAD heterogeneity; (4) absence of clinically applicable prognostic models integrating PLK1 expression.

To address these gaps, we systematically investigated PLK1’s biological and clinical significance in LUAD through the following approaches: (1) transcriptome-based pathway analysis: Leveraging RNA-seq data from The Cancer Genome Atlas (TCGA) and Gene Expression Omnibus (GEO) databases to identify PLK1-associated transcriptional networks; (2) TME interaction profiling: Quantifying immune cell infiltration and stromal activity using ssGSEA; (3) prognostic model construction: Developing a nomogram incorporating PLK1 expression and clinicopathological parameters to predict overall survival; (4) functional validation: Employing A549 cell models to evaluate the effects of PLK1 inhibition (*via* GSK461364) on proliferation, apoptosis, and cell cycle regulation. These findings establish PLK1 as a critical regulator of LUAD progression and a promising therapeutic target.

## Materials & Methods

### Data acquisition and preprocessing

This research employed Xiantao Academic (https://www.xiantao.love/) to conduct bioinformatics analysis and visualize data. The platform enabled extensive analyses such as pan-cancer analysis, differential expression assessments, clinical relevance evaluation, functional clustering, and related statistical analyses. Transcriptomic profiles and corresponding clinicopathological metadata for LUAD cohorts (TCGA-LUAD) were acquired through GDC portal (http://portal.gdc.cancer.gov) and UCSC XENA browser (http://xenabrowser.net/datapages/). STAR-aligned and Toil-pipeline normalized transcript-level TPM matrix has been used for downstream analysis (Vivian et al., 2017). Independent validation was performed using dataset GSE115002 from NCBI’s GEO repository. Representative immunohistochemical (IHC) images of PLK1 protein expression in normal lung tissue and lung adenocarcinoma have been obtained from Human Protein Atlas (HPA) (http://www.proteinatlas.org/) (Uhlén et al., 2015). Gene mutation and copy number alteration (CNA) data have been obtained from cBioPortal (http://www.cBioportal.org/) (De Bruijn et al., 2023). Transcriptomic data are log2(TPM + 1) transformed before analysis. Normal tissue samples and incomplete clinical annotations are excluded.

### Differential expression analysis

**Figure 1:**
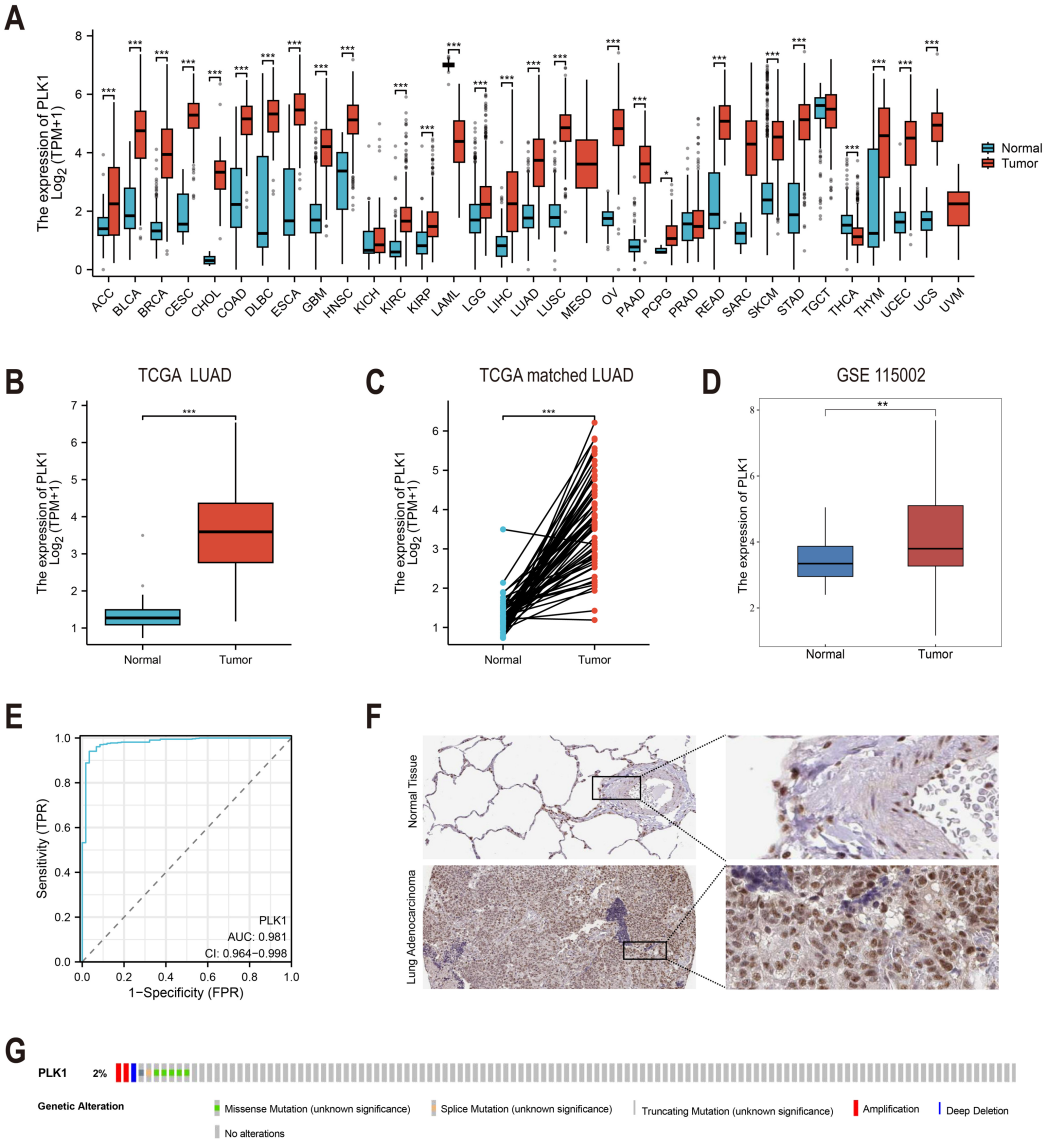
PLK1 expression profiling in LUAD. (A) Cross-database analysis of PLK1 transcript levels in malignant *vs.* non-neoplastic tissues (TCGA+GTEx). (B) Comparative quantification in LUAD specimens (*n* = 539) *versus* normal controls (*n* = 59). (C) Matched-pair evaluation of tumor-adjacent normal tissue pairs (*n* = 58). (D) Validation cohort analysis using GSE115002 dataset. (E) Diagnostic efficacy assessment *via* ROC curve analysis. (F) HPA validation of PLK1 protein expression. (G) Genomic alteration landscape from cBioPortal (OncoPrint). Significance thresholds: * *P* < 0.05, ** *P* < 0.01, *** *P* < 0.001.

To investigate the expression pattern of PLK1 (ENSG00000166851.15), we performed the following comparisons: Pan-cancer analysis (Fig. 1A): expression of PLK1 on TCGA and Genotype-Tissue Expression (GTEx) cohorts. Unpaired analysis (Fig. 1B): Tumor *vs.* normal LUAD samples using Wilcoxon rank sum test. Paired sample analysis (Fig. 1C): Matched tumor-normal LUAD samples using paired *t*-test. External validation (Fig. 1D): Analysis of GSE115002 using Wilcoxon test. Statistical computations were executed in R (v4.2.1) using stats, car, and ggplot2 packages.

### Diagnostic value assessment

The diagnostic accuracy of PLK1 was evaluated through time-dependent AUC assessment (Fig. 1E) using package pROC (v1.18.0). Curves and AUC values were visualized using ggplot2 (v3.4.4).

### Association with clinicopathological features

**Figure 2:**
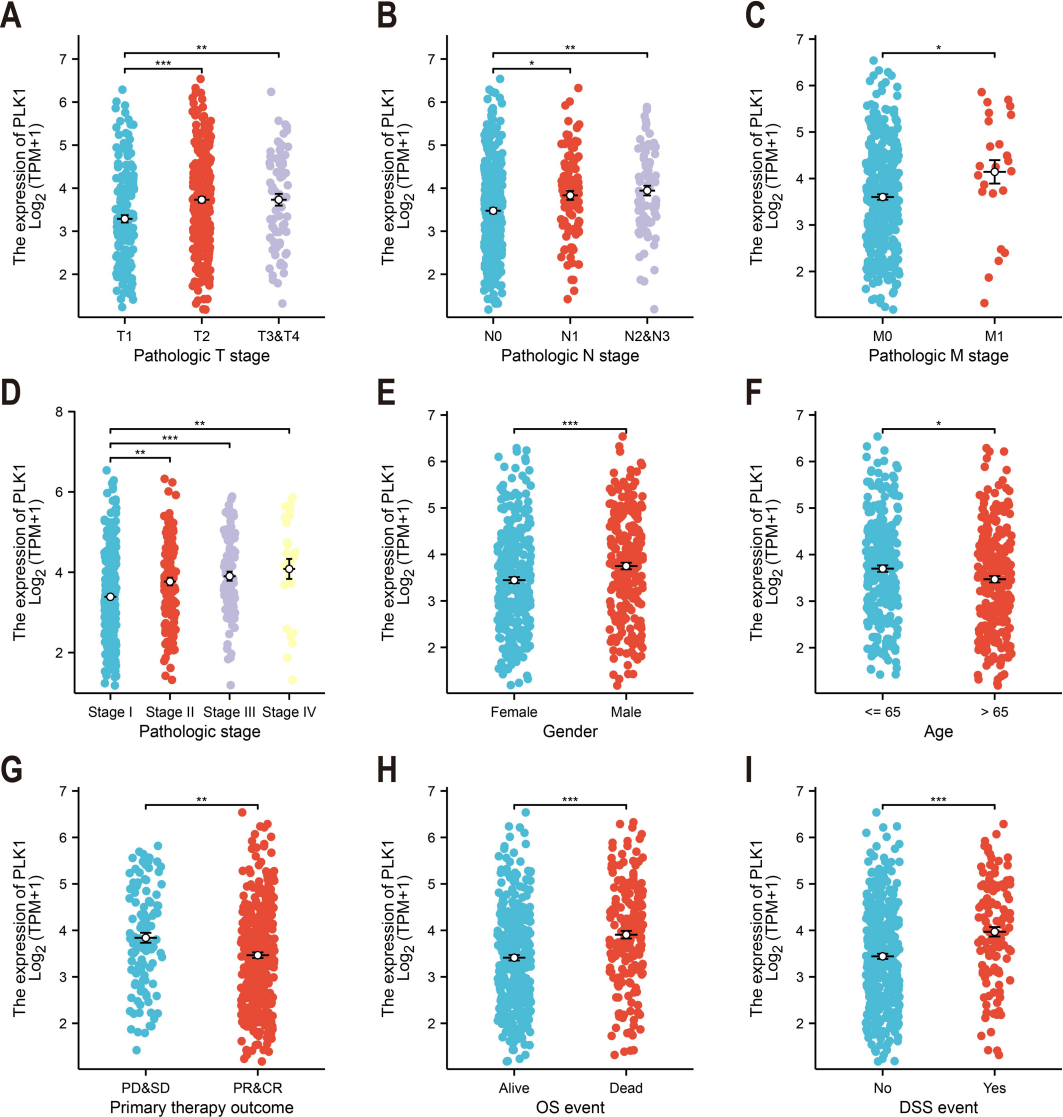
PLK1-clinicopathological correlations in TCGA-LUAD. (A) Pathological tumor staging (T). (B) Nodal metastasis status (N). (C) Distant metastasis occurrence (M). (D) Comprehensive disease staging. (E) Gender-based expression variance. (F) Age stratification (≤65 *vs* > 65 years). (G) Therapeutic response correlation. (H) Overall survival outcomes. (I) Disease-specific mortality events. Significance levels: * *P* < 0.05, ** *P* < 0.01, *** *P* < 0.001.

Association of PLK1 expression with Clinicopathological Features (*e.g.*, tumor stage, TNM classification, gender) was tested by Kruskal-Wallis test (Figs. 2A–2I). Relevant data were extracted from TCGA; incomplete cases were excluded from subgroup analysis.

### Survival analysis

**Figure 3:**
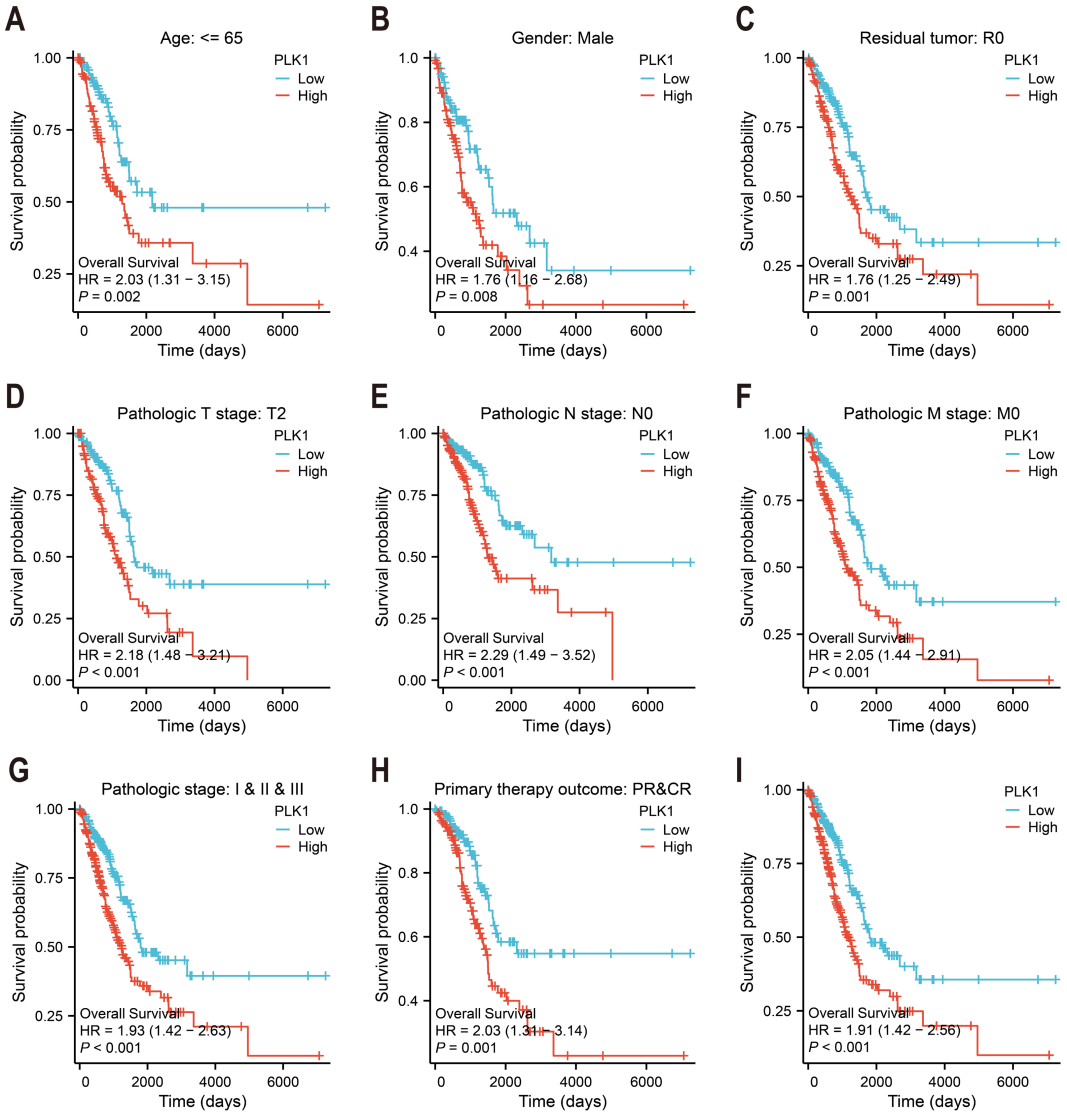
PLK1 prognostic value in LUAD cohorts. (A–H) Stratified survival analysis by clinicopathological parameters: age stratification, gender distribution, residual tumor status, tumor/node/metastasis (T/N/M) staging, comprehensive pathological staging, therapeutic response; (I) TCGA-derived overall survival correlation analysis. Curves generated using Kaplan–Meier methodology.

PLK1’s survival prediction capacity was quantified through Kaplan–Meier survival curves and univariate Cox proportional hazards regression (Figs. 3A–3I). Patients were categorized according to median expression level. Survival (v3.3.1) and survminer (v0.4.9) packages were used. Logrank test was used to evaluate statistical significance. Clinical follow-up data were supplemented by survival metadata from Liu et al. (2018).

### Nomogram construction

**Figure 4:**
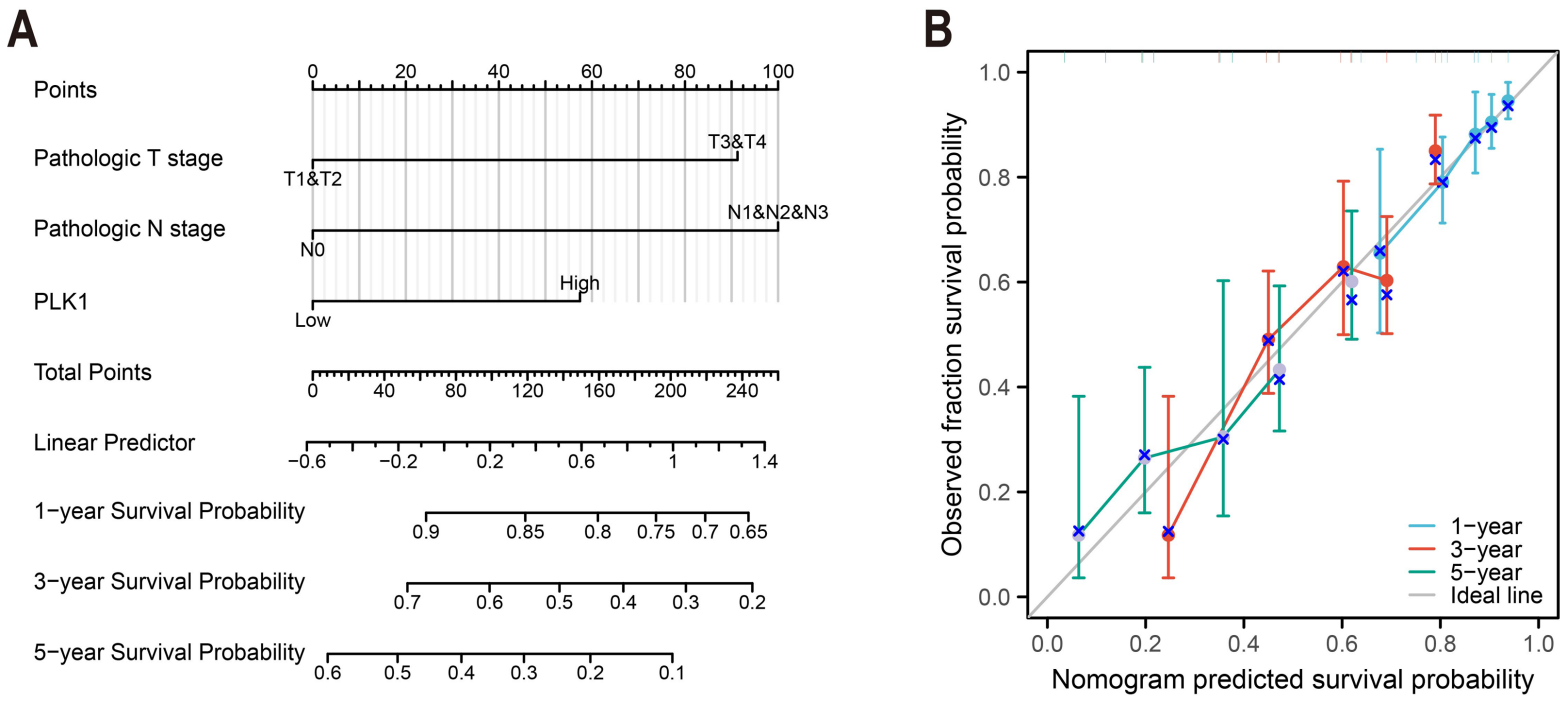
LUAD prognostic nomogram development. (A) Multivariable model integrating: Pathological T/N staging, PLK1 expression levels, predicting 1-/3-/5-year survival probabilities; (B) calibration performance evaluation: predicted *vs* observed outcomes, reference line (45° gray) indicates ideal calibration.

To construct a prognostic model of overall survival, optimal expression cutoff values were determined using surv_cutpoint function with bootstrap resampling (200 iterations, 80 samples each). Multivariable survival modeling was performed through proportional hazards regression to identify clinicopathologically independent predictors (Table 1). The prognostic prediction system was developed with the rms package (v6.3-0), and calibration plots were used for 1-, 3- and 5-year survival intervals (Fig. 4).

**Table 1:** Univariate and multivariate cox regression analyses of clinical characteristics associated with overall survival of LUAD in TCGA.

Characteristics	Total (N)	Univariate analysis		Multivariate analysis	
		HR (95% CI)	*P* value	HR (95% CI)	*P* value
Age	520				
≤65	257	Reference			
>65	263	1.216 (0.910–1.625)	0.186		
Gender	530				
Female	283	Reference			
Male	247	1.087 (0.816–1.448)	0.569		
Pathologic T stage	527				
T1&T2	461	Reference		Reference	
T3&T4	66	2.352 (1.614–3.426)	<0.001	2.134 (1.407–3.235)	<0.001
Pathologic N stage	514				
N0	345	Reference		Reference	
N1&N2&N3	169	2.547 (1.904–3.407)	<0.001	2.028 (1.435–2.867)	<0.001
Pathologic M stage	381				
M0	356	Reference		Reference	
M1	25	2.176 (1.272–3.722)	0.005	1.531 (0.855–2.742)	0.152
PLK1	530				
Low	267	Reference		Reference	
High	263	1.909 (1.423–2.562)	<0.001	1.692 (1.186–2.413)	0.004

### Differential gene expression and functional enrichment

**Figure 5:**
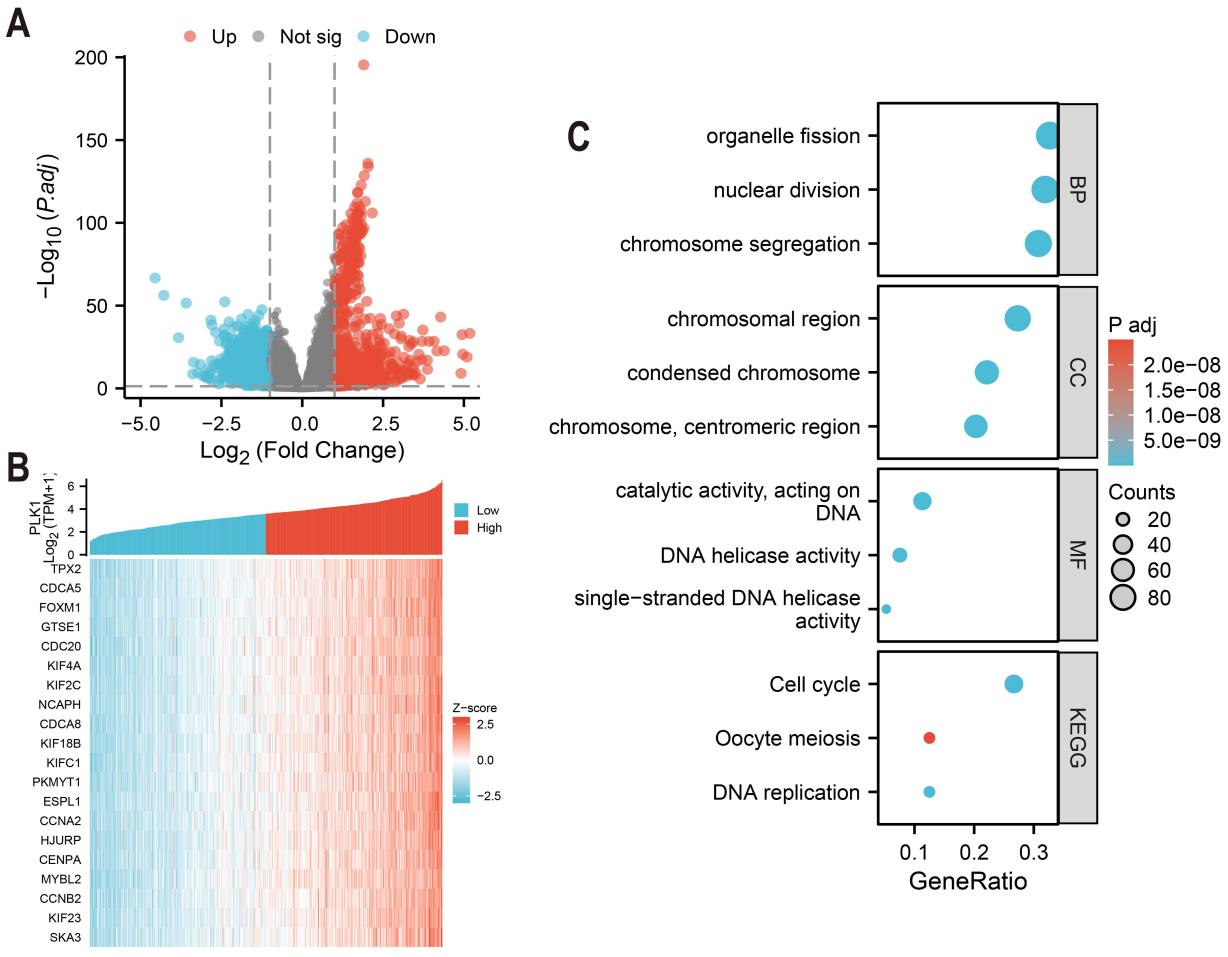
PLK1 functional network analysis in LUAD. (A) Transcriptomic landscape of PLK1-associated DEGs (high *vs* low expression); (B) expression profiling of top 20 PLK1-correlated genes; (C) Functional enrichment analysis: GO categories (BP/CC/MF), KEGG pathway mapping, visual parameters: Dot size = gene quantity; Color gradient = −log10(adj. *p*).

Differentially expressed genes (DEGs) of high and low PLK1 expression groups (top 50% *vs.* bottom 50%) were identified using DESeq2 (v1.36.0) with default parameters (Love, Huber & Anders, 2014). Genes with an adjusted *p*-value (FDR) < 0.05 and an absolute log2 fold change > 1 were defined as statistically significant. The analysis using edgeR (v3.38.2) on the same raw count data yielded consistent results (Fig. 5A). The top 20 genes strongly co-expressed with PLK1 were selected according to Spearman correlation and visualized using heatmap (Fig. 5B). GO and KEGG pathway enrichment analyses were performed using clusterProfiler (v4.4.4) (Yu et al., 2012), considering terms with an FDR <0.05 as significantly enriched. Gene ID annotation was performed using org.Hs.eg.db and visualisation with custom ggplot2 scripts (Fig. 5C).

### Immune cell infiltration study

**Figure 6:**
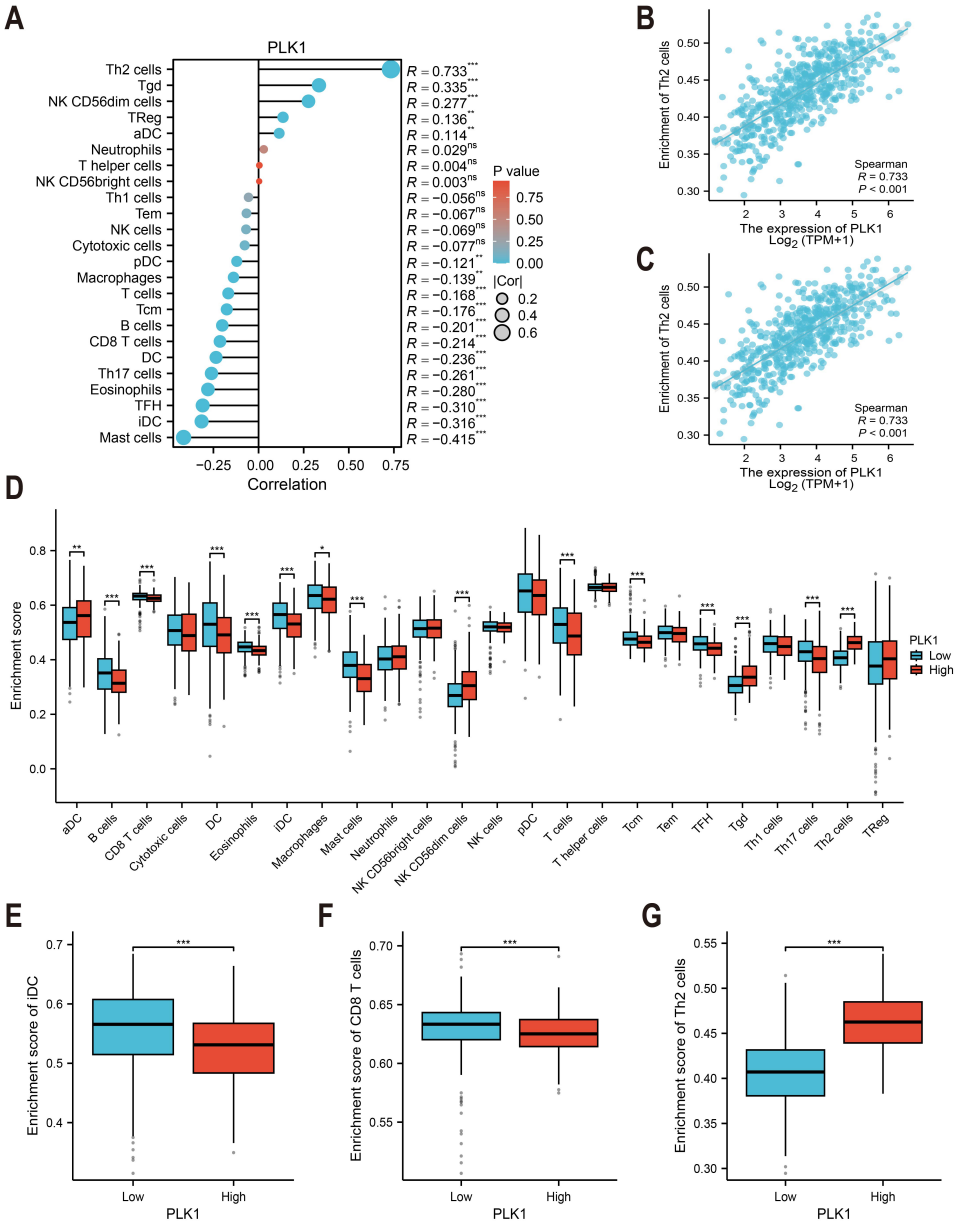
PLK1-immune microenvironment interactions in LUAD. (A) Spearman’s correlation matrix: PLK1 *vs* immune infiltrates; (B,C) bivariate analysis with Th2 cells (B) and immature dendritic cells (iDCs) (C); (D) immune cell composition comparison (high *vs* low PLK1); (E–G) differential infiltration quantification: iDCs (E); CD8^+^ T lymphocytes (F); Th2 cells (G). Significance thresholds: * *P* < 0.05, ** *P* < 0.01, *** *P* < 0.001.

Immune cell infiltration was estimated using ssGSEA with the GSVA package (v1.46.0) (Fig. 6A) (Hänzelmann, Castelo & Guinney, 2013). The immune cell gene sets (*n* = 24) were selected from Bindea et al. (2013). The correlation between PLK1 expression and immune cell abundance (*e.g.*, Th2 cells and immature dendritic cells) was assessed using Spearman rank correlation (Figs. 6B, 6C). Immune infiltration scores were compared between PLK1-high and PLK1-low using Wilcoxon rank sum test (Figs. 6D–6G). All plots were generated using ggplot2.

### External validation analysis

The robustness of our findings was assessed through external validation using independent data and tools. The association between PLK1 expression and overall survival in LUAD was examined using the Kaplan–Meier plotter online tool (https://kmplot.com/analysis/index.php?p=home), which aggregates data from multiple GSE datasets within the GEO repository (Posta & Gyorffy, 2025). Furthermore, the GSE115002 dataset was utilized to reconfirm key associations, including the link between PLK1 expression and clinical stage, the functional enrichment profiles of PLK1-co-expressed genes, and the correlation between PLK1 expression and immune cell infiltration levels *via* ssGSEA. Analytical methods were consistent with those applied in the primary analysis. The corresponding data have been provided in the Supplemental Information.

### Cell culture and PLK1 specific inhibitor treatment

The A549 cell line, derived from human lung adenocarcinoma was generously provided by Prof. Lijun Shi from the First Affiliated Hospital of Guangxi Medical University. The cell line was authenticated by short tandem repeat (STR) profiling within the last six months and routinely tested to confirm the absence of mycoplasma contamination. A549 cells were cultured in RPMI 1640 medium (cat.no. 61870036; Gibco) with 10% fetal bovine serum (FBS, cat.no. A5256701; Gibco) at 37 °C with 5% CO_2_. Cells were maintained at 37 °C in a humidified atmosphere with 5% CO_2_ and routinely passaged at 80–90% confluence using 0.25% Trypsin-EDTA (cat. no. 25300054; Gibco). All cell culture procedures were performed under standard sterile conditions. In order to inhibit PLK1 activity we used selective small-molecule inhibitor GSK461364 (Cat. no. G873280, CAS: 929095-18-1; Macklin Biochemical Co., Shanghai, China). This ATP-competitive inhibitor exhibits high selectivity for PLK1, with a half-maximal inhibitory concentration (IC50) of approximately 2.2 nM and more than 390-fold selectivity over PLK2 and PLK3 (Olmos et al., 2011). Consequently, based on preliminary dose–response experiments and published pharmacokinetic data, a working concentration of 40 nM GSK461364 was selected for all subsequent functional assays to ensure efficacy within the selective window for PLK1 inhibition (Gilmartin et al., 2009). Dimethyl sulfoxide (DMSO, cat.no. A68908; Innochem) at a final concentration of 0.1% was used in an equivalent volume as the vehicle control. All downstream assays were performed immediately after treatment. This pharmacological inhibition approach (rather than siRNA-mediated gene silencing) was chosen to recapture therapeutic drug effects and to enhance its translational relevance for future studies.

### Cell proliferation assessment (CCK-8)

The proliferative potential of A549 cells was quantitatively analyzed using the CCK-8 colorimetric assay (cat.no. C0037; Beyotime). After 12-hour pre-culture in 96-well plates (1.5 × 10^3^ cells/well), the medium was replaced with fresh solutions containing: Vehicle control (0.1% DMSO) and 40 nM GSK461364. At designated time points (0–4 days post-treatment), 10 μL CCK-8 reagent was added to each well and incubated for 1 h at 37 °C. Optical density measurements at 450 nm were acquired using a GloMax Discover multimode microplate reader (Promega Corporation).

### Clonogenic survival analysis

A549 cells (1 × 10^3^ cells/well) seeded in 6-well plates received 24-hours GSK461364 treatment (40 nM), followed by PBS washing and 6-day maintenance in drug-free medium for clonogenic assessment. Post-treatment procedures included: (1) PBS washing to remove non-adherent cells, (2) fixation with 4% paraformaldehyde (15 min, cat. no. P00999; Beyotime), and (3) staining with 0.5% crystal violet solution (15 min, cat.no. C0121). ImageJ software was used for quantifying Colony formation assay.

### Apoptosis analysis

To induce apoptosis, A549 cells (5 × 10^5^ cells/well) were treated with 40 nM GSK461364 or vehicle control (0.1% DMSO) for 24 h. Quantitative apoptosis assessment was then conducted using the FITC Annexin V/PI dual-staining kit (C1062M; Beyotime Biotechnology). Following treatment, cells were harvested *via* trypsinization (0.25% trypsin-EDTA, cat.no. 25200056; Gibco). Cell pellets were resuspended in binding buffer and dual-stained with Annexin V-FITC/PI (15 min, dark). Accuri C6 flow cytometer (BD Biosciences) was used for detection and flowJo software 10.9.0 was used for analysis.

Gating strategy: The intact cell population was first identified on an FSC-A *vs.* SSC-A dot plot to exclude debris. Single cells were then selected using an FSC-H *vs.* FSC-A gate to exclude doublets. Apoptotic populations were finally quantified on an Annexin V-FITC *vs.* PI dot plot.

### Cell cycle assay

A549 cells (5 × 10^5^/well) in 6-well plates were treated with 40 nM GSK461364 or vehicle control for 24 h. Floating and adherent cells (harvested *via* 0.25% trypsin digestion) were pooled, fixed in 75% ethanol (−20 °C, 16 h), then subjected to RNase A treatment (100 µg/mL, 37 °C, 30 min) prior to PI staining (50 µg/mL, 15 min dark incubation). Cell cycle distribution was quantified using a BD Accuri C6 flow cytometer analyzed with FlowJo v10.9.0.

Gating strategy: After gating on the intact single-cell population (FSC-A *vs.* SSC-A) and excluding doublets (FSC-H *vs.* FSC-A), the DNA content of single cells was analyzed on a PI-A histogram. The cell cycle phase distribution (G0/G1, S, G2/M) was modeled and quantified using the built-in “Cell Cycle” analysis module in FlowJo.

### Western blotting

To assess the effects of PLK1 inhibition on cell cycle regulatory proteins, A549 cells were treated with 40 nM GSK461364 or vehicle control (0.1% DMSO) for 24 h prior to protein extraction. Cellular proteins were lysed in RIPA buffer supplemented with protease inhibitor cocktail. After centrifugation (12,000× g, 15 min), protein concentrations were quantified spectrophotometrically (NanoDrop). 25 µg of protein per lane was separated on 10% SDS-polyacrylamide gels and electrophoretically transferred to PVDF membranes (beyotime, cat.no. FFP24). The transfer was conducted at 100 V for 60 min on ice. Membranes were blocked with QuickBlock buffer for 15 min at room temperature prior to overnight incubation (4 ° C) with primary antibodies: Cyclin B1 (cat. no. ab181593, clone EPR17060, 1:2000; Abcam), CDK1 (cat.no. ab32094, clone YE324, 1:5000; Abcam), and β-actin (cat.no. 66009-1-lg, 2D4H5, 1:10,000; Proteintech). HRP-conjugated goat anti-mouse (cat.no. P0946, 1:1000; Beyotime) or goat anti-rabbit (#P0948, 1:1000; Beyotime) secondary antibodies were applied for 1 h at RT, and proteins were detected using an ECL substrate.

### Statistical analysis

Differences in PLK1 expression were assessed through Wilcoxon tests or paired *t*-tests. Survival was evaluated using Kaplan–Meier/log-rank tests, while associations were analyzed using Spearman’s correlation. Experimental comparisons utilized *t*-tests/ANOVA after confirming normality. Benjamini–Hochberg FDR correction was applied as needed (* *p* < 0.05, ** *p* < 0.01, *** *p* < 0.001, **** *p* < 0.0001). Data from triplicate experiments are presented as mean ± SEM and were analyzed with GraphPad Prism v10 (experimental data) and R v4.2.1 (bioinformatics data) to ensure methodological consistency.

## Results

### Upregulation of PLK1 expression in LUAD tissues

Analysis of TCGA and GTEx databases revealed differential PLK1 expression across various cancer types (Fig. 1A). In LUAD patients, tumor tissues demonstrated significantly elevated PLK1 expression compared to adjacent normal tissues (Figs. 1B, 1C). Validation using the GSE115002 dataset (52 paired samples) confirmed higher PLK1 levels in tumor tissues (*p* < 0.001, Fig. 1D). ROC curve analysis indicated high diagnostic accuracy of PLK1 expression with an AUC of 0.981 (0.964–0.998) (Fig. 1E). IHC images from the HPA database corroborated enhanced PLK1 protein expression in LUAD specimens (Fig. 1F). Genomic alteration analysis *via* cBioPortal showed PLK1 amplifications, deep deletions, and missense mutations occurring in <2% of cases (Fig. 1G). These multi-platform findings substantiate PLK1 overexpression in LUAD.

### Association between PLK1 expression and clinicopathological features in LUAD

Analysis of TCGA data revealed significant correlations between PLK1 expression levels and nine clinicopathological parameters (Fig. 2). Elevated PLK1 expression showed strong associations with advanced T stage (*p* < 0.01, Fig. 2A), N stage (*p* < 0.01, Fig. 2B), M stage (*p* < 0.05, Fig. 2C), and higher pathological stage (*p* < 0.01, Fig. 2D). Significant relationships were also observed with gender (*p* < 0.001, Fig. 2E), age (*p* < 0.05, Fig. 2F), primary therapy outcome (*p* < 0.01, Fig. 2G), overall survival events (*p* < 0.001, Fig. 2H), and disease-specific survival events (*p* < 0.001, Fig. 2I). These findings indicate that PLK1 is not only upregulated in LUAD but also significantly associated with key clinicopathological features.

### Elevated PLK1 levels are linked to unfavorable outcomes in LUAD patients

Kaplan–Meier survival analysis of TCGA data stratified by median PLK1 expression revealed significantly worse overall survival in high-expression patients (HR = 1.91, *p* < 0.001; Fig. 3I). Subgroup analyses demonstrated consistent prognostic associations across multiple clinical contexts: younger patients (<65 years) showed heightened risk (HR = 2.03, *p* = 0.002; Fig. 3A), as did male subjects (HR = 1.76, *p* = 0.008; Fig. 3B) and those achieving R0 resection (HR = 1.76, *p* = 0.001; Fig. 3C). Elevated PLK1 expression maintained predictive value across tumor stages—T2 (HR = 2.18, *p* < 0.001; Fig. 3D), N0 (HR = 2.29, *p* < 0.001; Fig. 3E), and M0 (HR = 2.05, *p* < 0.001; Fig. 3F)—as well as pathological stages I–III (HR = 1.93, *p* < 0.001; Fig. 3G). Notably, patients with complete/partial treatment response (CR/PR) still exhibited increased mortality risk (HR = 2.03, *p* = 0.002, Fig. 3H). These stratified findings establish PLK1 overexpression as a multi-contextual prognostic biomarker in LUAD.

### PLK1 as independent prognostic determinant in LUAD survival

Cox regression analyses incorporating age, gender, and TNM stage parameters identified elevated PLK1 expression as an independent risk factor for reduced overall survival (HR = 1.692, 95% CI [1.186–2.413], *p* = 0.004). Multivariate assessment confirmed significant prognostic contributions from advanced T stage (HR = 2.134, 95% CI [1.407–3.235], *p* < 0.001) and nodal involvement (N stage: HR = 2.028, 95% CI [1.435–2.867], *p* < 0.001), as detailed in Table 1. These findings establish PLK1 overexpression as a distinct biological predictor of clinical outcomes independent of conventional staging parameters.

### PLK1-incorporated nomogram for LUAD prognostication

A predictive nomogram integrating three multivariate-validated prognostic determinants—T stage, N stage, and PLK1 expression—was developed to estimate 1-, 3-, and 5-year survival probabilities (Fig. 4A). Calibration analysis demonstrated strong concordance between model predictions and observed outcomes across all timepoints (Fig. 4B). The model achieved a concordance index of 0.692 (95% CI [0.668–0.716]), indicating moderate discriminative capacity. Comparative assessment revealed superior predictive performance of this composite model over individual prognostic parameters (Fig. 4B), establishing its clinical utility for stratified survival prediction in LUAD management.

### Functional characterization of PLK1-associated genes in LUAD

Comparative analysis of high *vs* low PLK1 expression groups using DESeq2/edgeR identified 1,786 protein-coding DEGs (—log_2_FC— > 1, adj.*p* < 0.05), comprising 912 upregulated and 874 downregulated genes (Fig. 5A). Pearson correlation analysis revealed 275 strongly PLK1-associated genes (*r* > 0.6, adj.*p* < 0.05), with top 20 positive correlations visualized (Fig. 5B). Functional enrichment of these co-expressed genes demonstrated significant involvement in nuclear division processes (GO: BP), chromosomal organization (GO: CC), and DNA catalytic activities (GO: MF), along with cell cycle regulation pathways (KEGG) (Fig. 5C). Quantitative enrichment metrics identified 340 BP terms, 72 CC terms, 40 MF terms, and 16 KEGG pathways (adj.*p*/q < 0.05), with detailed annotations cataloged in Table S1.

### PLK1 expression is correlated with altered immune cell infiltration in LUAD

TCGA-based analysis revealed PLK1 expression positively correlated with Th2 cells (*r* = 0.733, *p* <0.001, Fig. 6B), T*γδ* cells, NK CD56dim cells, regulatory T cells (Tregs), and activated dendritic cells (aDCs). Conversely, inverse associations were observed with mast cells, immature dendritic cells (iDCs: r = −0.316, *p* < 0.001, Fig. 6C), follicular helper T cells (TFH), eosinophils, Th17 cells, conventional dendritic cells (cDCs), CD8+ T cells, B cells, central memory T cells (Tcm), total T cells, macrophages, and plasmacytoid dendritic cells (pDCs) (Fig. 6A). Comparative analysis between PLK1-high/low groups demonstrated significant differential infiltration of Th2 cells, iDCs, and CD8+ T cells (*p* < 0.05; Figs. 6D–6G). These correlative findings are consistent with a model in which high PLK1 expression may contribute to an immunosuppressive TME, potentially through coordinated associations with Th2 polarization, impaired dendritic cell maturation, and reduced cytotoxic T cell infiltration.

### External validation of key findings

To reinforce the robustness of our findings, we validated the core results in independent external datasets. Initial analysis of aggregated GEO cohorts confirmed that high PLK1 expression was significantly associated with poorer overall survival (pooled HR = 1.95, 95% CI [1.64–2.33], *p* < 3.3e-14). Subsequent analysis in the GSE115002 validation cohort consistently recapitulated our primary findings: high PLK1 expression was significantly correlated with advanced clinical stage (*p* = 0.033, Cohen’s d = −0.771, indicating a medium effect size toward higher stage), and PLK1-co-expressed genes were again predominantly enriched in cell cycle-related pathways. Furthermore, the significant association between high PLK1 expression and an immunosuppressive tumor microenvironment was confirmed by ssGSEA. These consistent results across independent cohorts substantially strengthen the generalizability of our conclusions. Detailed results are presented in Fig. S1.

### PLK1 inhibition suppresses oncogenic activity in A549 cells

**Figure 7:**
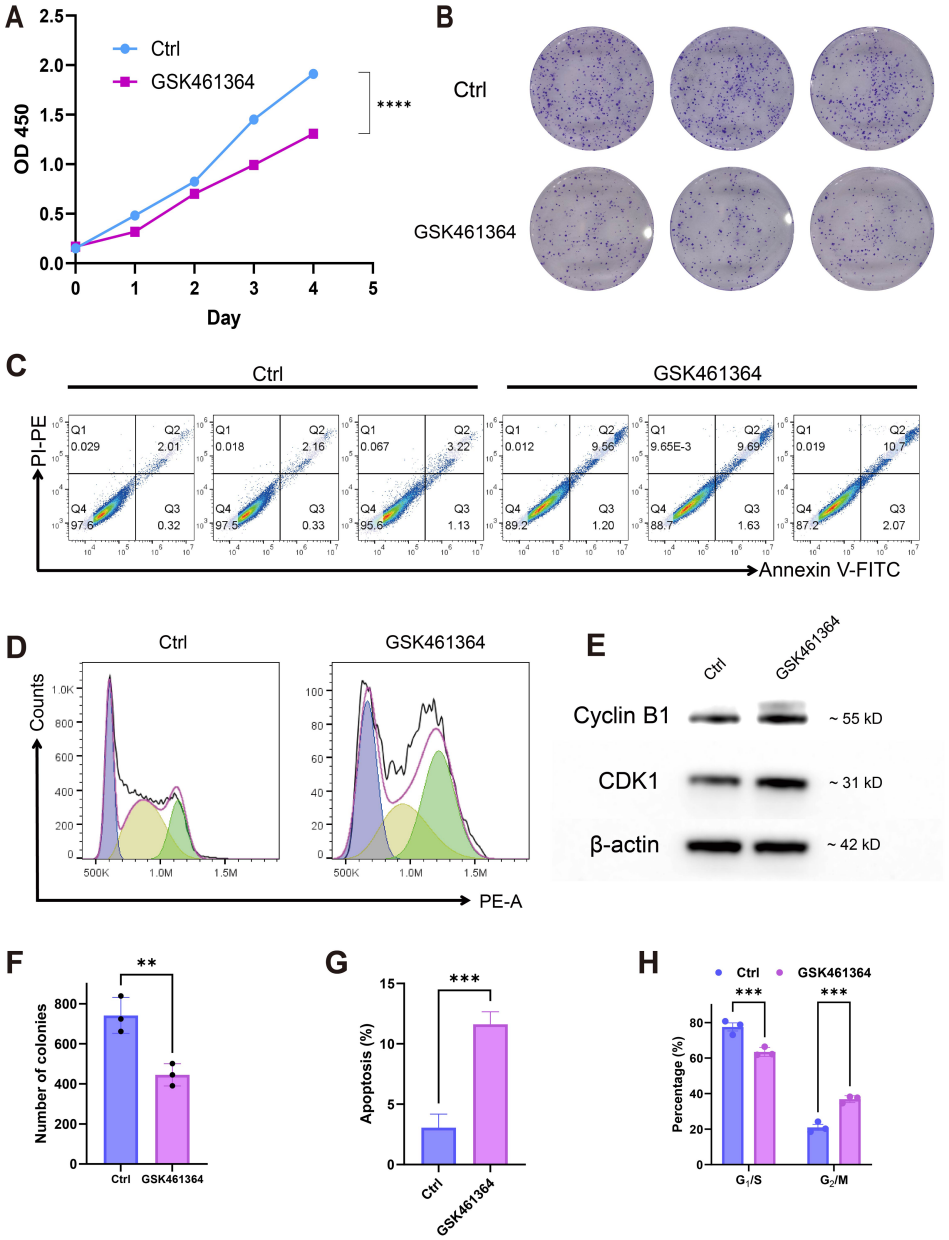
PLK1 inhibition phenotypic effects in A549. (A) Proliferation suppression (CCK-8 assay); (B, F) clonogenic impairment: representative colonies (B), quantitative colony counts (F); (D, E) apoptosis induction: flow cytometric profiles (C), apoptotic cell quantification (G); (D, H) cell cycle modulation: phase distribution patterns (D), G2/M arrest quantification (H); (E) cell cycle regulators: Cyclin B1/CDK1 protein expression, β-actin normalization; data: Mean ± SD (triplicates); Significance: *P < 0.05, **P ¡ 0.01, ***P < 0.001, ****P < 0.0001 *vs* control.

Pharmacological PLK1 inhibition significantly attenuated A549 proliferative capacity, evidenced by reduced CCK-8 viability (Fig. 7A) and diminished colony formation (Figs. 7B, 7F). Cell cycle profiling revealed G2/M phase arrest (Figs. 7D, 7H) accompanied by elevated apoptosis rates (Fig. 7C,7G). Western blot analysis demonstrated concomitant accumulation of Cyclin B1 and CDK1 (Fig. 7E), aligning with prior pathway enrichment findings implicating PLK1 in cell cycle regulation (Fig. 5C). These multimodal findings establish PLK1 as a critical regulator of oncogenic phenotypes in LUAD through cell cycle modulation and inhibiting cell survival.

## Discussion

PLK1 has been shown to have carcinogenic properties in many cancers; however, its role in the pathology of LUAD and its relationship with clinical features are rarely reported. In this paper, we aim to confirm PLK1’s role in LUAD and provide more evidence for potential therapeutic targets. We investigated the carcinogenic role of PLK1 in LUAD by combining bioinformatics and cell validation. Our transcriptome-based analysis revealed three key findings: (1) PLK1 overexpression correlates with advanced clinicopathological stages (T/N/M) and poor survival outcomes; (2) PLK1 co-expressed genes are enriched in cell cycle regulation networks; (3) high PLK1 expression is associated with an immunosuppressive tumor microenvironment characterized by Treg infiltration and CD8+ T cell exclusion. To validate these findings, cell experiments showed that treatment of A549 cells with GSK461364 inhibited cell proliferation, arrested the cell cycle in the G2/M phase, and increased apoptosis. Collectively, these results suggest that PLK1 may be a potential prognostic marker and therapeutic target for LUAD patients.

PLK1, a pivotal member of Polo-like kinases (PLKs) family located on chromosome 16p12.2, orchestrates genomic stability through the regulation of cell cycle progression, DNA repair mechanisms, and mitotic checkpoints (Gheghiani et al., 2021; Gelot et al., 2023; Conti et al., 2024). Its overexpression is commonly observed in various cancers, such as breast, prostate, and ovarian malignancies, correlating with unfavorable prognosis (Zhang et al., 2021; Wang et al., 2022; Gao et al., 2024; Stebbing & Bullock, 2024). Consistent with these reports, our findings in LUAD tissues confirm the high expression of PLK1 (Figs. 1B–1D, 1F), particularly in advanced pathological stages (Figs. 2A–2D). Functional studies demonstrated PLK1 inhibition-mediated suppression of A549 proliferative capacity (colony formation), cell cycle arrest, and apoptosis induction (Fig. 7), indicating its potential involvement in LUAD development and progression. Clinical analyses established PLK1’s prognostic significance: high expression correlated with reduced therapeutic response rates, diminished overall/disease-specific survival (Figs. 2G–2I, 3), and independent risk factor status (Table 1). Diagnostic utility was evidenced by ROC curve analysis (Fig. 1E), while the developed nomogram (Fig. 4) provides clinical decision-making support through survival probability stratification (Fig. 4).

A central finding of our study is the elucidation of the mechanism underlying PLK1 inhibitor-induced cell cycle arrest. Functional enrichment analysis of genes co-expressed with PLK1 revealed that these genes are involved in cell cycle regulation (Fig. 5). Experimentally, PLK1 inhibition increased G2/M arrest in A549 cells (Figs. 7D, 7H) and accumulation of Cyclin B1 and CDK1 proteins (Fig. 7E). This observation is explained by a key mechanistic insight: PLK1 inhibition prevents the activation of the anaphase-promoting complex/cyclosome (APC/C), which is essential for the targeted degradation of Cyclin B1. This results in the paradoxical accumulation of both Cyclin B1 and CDK1. However, the CDK1 kinase becomes improperly regulated, leading to aberrant mitotic entry and ultimately triggering mitotic catastrophe—a well-documented, paradoxical mechanism of PLK1 inhibition where the accumulation of pro-mitotic proteins culminates in cell death arrest (Belur Nagaraj et al., 2018; Chapagai et al., 2025; Gobran et al., 2025).

Building upon prior bioinformatic findings linking PLK1 expression to tumor staging and immune infiltration patterns (B lymphocytes, CD4+ T cells, macrophage populations) (Li et al., 2021), our comprehensive analysis reveals PLK1-overexpressing LUAD specimens demonstrate an immunosuppressive microenvironment characterized by elevated regulatory T cells (Tregs) and Th2 polarization, concurrent with diminished infiltration of immature dendritic cells (iDCs), conventional dendritic cells (cDCs), and cytotoxic CD8+ T lymphocytes (Fig. 6). This aligns with known mechanisms: Tregs suppress effector T cell activation (Iglesias-Escudero, Arias-González & Martínez-Cáceres, 2023), while DCs are essential for antigen presentation (Chen et al., 2024). The immunosuppressive TME observed in PLK1-high tumors likely results from complex interactions between tumor cells, immune cells, and chemokine networks, a recognized hallmark of cancer immunotherapy response (Nagarsheth, Wicha & Zou, 2017; Hegde & Chen, 2020). For instance, the CXCL12-CXCR4 axis is known to recruit Tregs and promote an immune-excluded phenotype (Hegde & Chen, 2020). In our context, PLK1-driven CXCL2 secretion (Kong et al., 2024) may similarly facilitate the recruitment of immunosuppressive cells. Conversely, the lack of cytotoxic T-cell infiltration may be attributable to the epigenetic silencing of T helper 1 (TH1)-type chemokines like CXCL9 and CXCL10, which is known to create a ’cold’ tumor immune contexture (Nagarsheth, Wicha & Zou, 2017). Notably, Kong et al. (2024) demonstrated *via* single-cell RNA sequencing that PLK1 promotes M2 macrophage polarization *via* CXCL2 secretion and reduces MHC-II expression in antigen-presenting cells. Similarly, Xu et al. found that PLK1-mediated FoxM1 phosphorylation drives macrophage polarization, facilitating immune evasion (Xu et al., 2023). These findings collectively suggest a potential synergy between PLK1 inhibitors and immune checkpoint inhibitors. Supporting this, Zhang et al. (2022a) and Zhang et al. (2022b) showed that PLK1 inhibition activates the NF-κB pathway, upregulating PD-L1, while Reda et al. (2022) developed a nanoparticle-based therapy co-targeting PLK1 and PD-L1.

PLK1 inhibitor therapy has demonstrated efficacy in multiple cancer types. For instance, Volasertib suppresses tumor growth by inducing G2/M phase arrest and apoptosis (Zhang et al., 2022a; Zhang et al., 2022b; Garlapati et al., 2023; Wei et al., 2023)). However, resistance mechanisms—particularly p53-mediated survival in wild-type cells—have been reported (Yim & Erikson, 2014). Additionally, the TME may compromise efficacy *via* TGF-β secretion Barbosa Rabago et al., 2021) or EMT-driven drug efflux (Celià-Terrassa & Kang, 2024; Wu et al., 2024). Notably, while PLK1 inhibitor-induced G2/M arrest is a well-established radiosensitization strategy (Zhao et al., 2024), caution is warranted in radioimmunotherapy combinations as concurrent PD-L1 upregulation (Reda et al., 2022) may counteract radiation-induced immunogenic cell death.

While this investigation significantly advances our understanding of PLK1’s multifaceted role in LUAD pathogenesis and immune modulation, it is crucial to contextualize these findings within its inherent limitations. Our study establishes a correlative, rather than causal, link between PLK1 overexpression and an immunosuppressive tumor microenvironment (TME), as inferred from transcriptomic analyses. Although our findings and existing literature suggest a plausible biological mechanism, direct experimental evidence for PLK1’s role in modulating immune cell recruitment or function in LUAD remains to be established.

A second methodological consideration is the absence of direct molecular confirmation of target engagement(*e.g.*, *via* Western blot analysis of PLK1 protein levels post-GSK461364 treatment). Nevertheless, the specificity of the observed phenotypes—G2/M arrest and apoptosis—is robustly supported by the high selectivity profile of the inhibitor (IC_5__0_ = 2.2 nM; >390-fold over PLK2/3), the concordance of these outcomes with the established mechanism of PLK1 inhibition, and the use of a concentration (40 nM) rigorously chosen to operate within the selective window for PLK1.

Beyond these mechanistic constraints, the generalizability of our findings may be influenced by the reliance on a Kirsten rat sarcoma (KRAS)-mutant cellular model (A549), which does not fully capture the genomic heterogeneity of LUAD. This implies that PLK1 dependency and therapeutic vulnerability could vary among major molecular subtypes. Given the central role of cell cycle dysregulation uncovered in our study, it is plausible that subtypes driven by oncogenes which heavily rely on robust cell proliferation (*e.g.*, KRAS-mutant) may be particularly susceptible to PLK1 inhibition. Conversely, the association between high PLK1 expression and an immunosuppressive tumor microenvironment, as demonstrated in our data, suggests that PLK1 inhibitors might synergize with immunotherapies in immune-“cold” subtypes, such as those with STK11 mutations. These hypotheses represent critical considerations for future patient stratification strategies. Furthermore, the transcriptomic inference of an immunosuppressive TME lacks experimental validation of direct PLK1-immune cell interactions. Other considerations include potential selection bias from retrospective clinical data and the oversimplification of excluding other critical TME components, such as cancer-associated fibroblasts.

Looking ahead, addressing these limitations provides a clear strategic roadmap for clinical translation. To definitively test causality and establish PLK1’s role in immune modulation, future studies should employ co-culture systems, PLK1 modulation in immunocompetent animal models, or analysis of paired patient samples pre- and post-PLK1 inhibitor treatment. The logical next steps involve utilizing more physiologically relevant models, such as patient-derived xenografts encompassing diverse LUAD subtypes, to validate the efficacy of PLK1 inhibition across different genomic backgrounds. Complementing this, the application of high-resolution techniques like single-cell RNA sequencing will be essential to deconvolute the immunosuppressive landscape and identify resistant cellular subpopulations (Kim et al., 2020).

Finally, exploring rational combination therapies—for instance, pairing PLK1 inhibitors with PARP inhibitors to exacerbate DNA damage or with immune checkpoint blockers to overcome immune evasion—while meticulously optimizing treatment sequencing, will be paramount for developing effective personalized therapeutic strategies (Siraj et al., 2023).

## Conclusions

We demonstrate that PLK1 overexpression serves as a significant independent prognostic indicator in LUAD, correlated with an immunosuppressive TME and driven by cell cycle dysregulation. This study confirms PLK1’s dual function as both a strong biomarker and a potential therapeutic target in LUAD.

## Supplemental Information

10.7717/peerj.20618/supp-1 Supplemental Information 1 Summary of the GO term and KEGG pathway details for PLK1 co-expression enrichment analyses

10.7717/peerj.20618/supp-2 Supplemental Information 2 Raw data for bioinformatics analysis and cellular experiments

10.7717/peerj.20618/supp-3 Supplemental Information 3 Data for External Validation Analysis

10.7717/peerj.20618/supp-4 Supplemental Information 4 External Validation of PLK1’s Prognostic and Functional Role in Independent LUAD Cohorts (A) Kaplan–Meier overall survival analysis of LUAD patients stratified by PLK1expression using aggregated GEO datasets (Pooled HR = 1.95, 95% CI [1.64–2.33], logrank *p* = 3.3e-14). (B) Co-expression heatmap of PLK1with top 20 positively/negatively correlated genes (GSE115002 cohort). (C) PLK1expression comparison between stage I–III and stage IV tumors (Wilcoxon rank-sum test, *p* = 0.033; Cohen’s d = − 0.771). (D) Immune cell infiltration profiles stratified by PLK1expression levels (ssGSEA algorithm). (E, F) Functional enrichment analysis of PLK1-co-expressed genes: GO biological processes (E) and KEGG pathways (F). Dot size represents gene count; color intensity indicates −log10 (adjusted *p* value).

## Additional Information and Declarations

### Data Availability

The following information was supplied regarding data availability: The bioinformatics analyses and cellular experiments raw data are available in the Supplemental Files. The Western Blotting Raw Data of Cell Cycle Arrest data is available at figshare: You, LuKuan (2025). Western Blotting Raw Data of Cell Cycle Arrest. figshare. Figure. https://doi.org/10.6084/m9.figshare.29369390.v4
The Flow Cytometry Raw Data of cell cycle arrest data is available at figshare: You, LuKuan (2025). Flow Cytometry Raw Data of cell cycle arrest. figshare. Dataset. https://doi.org/10.6084/m9.figshare.29369228.v1
The Flow Cytometry Raw data of Apoptosis data is available at figshare: You, LuKuan (2025). Flow Cytometry Raw data of Apoptosis. figshare. Dataset. https://doi.org/10.6084/m9.figshare.29369225.v1.
