# Supplementary material for: PLK1 overexpression as a dual-role biomarker and therapeutic vulnerability in pulmonary adenocarcinoma
Source: PeerJ. 2026 Jan 15;14:e20618. doi: 10.7717/peerj.20618 (PMC12812279; doi:10.7717/peerj.20618)
Supplement: Supplemental Information 2 [file peerj-14-20618-s002.tar › additional files/Figure 1F Expression of PLK1 in lung cancer - The Human Protein Atlas.pdf]

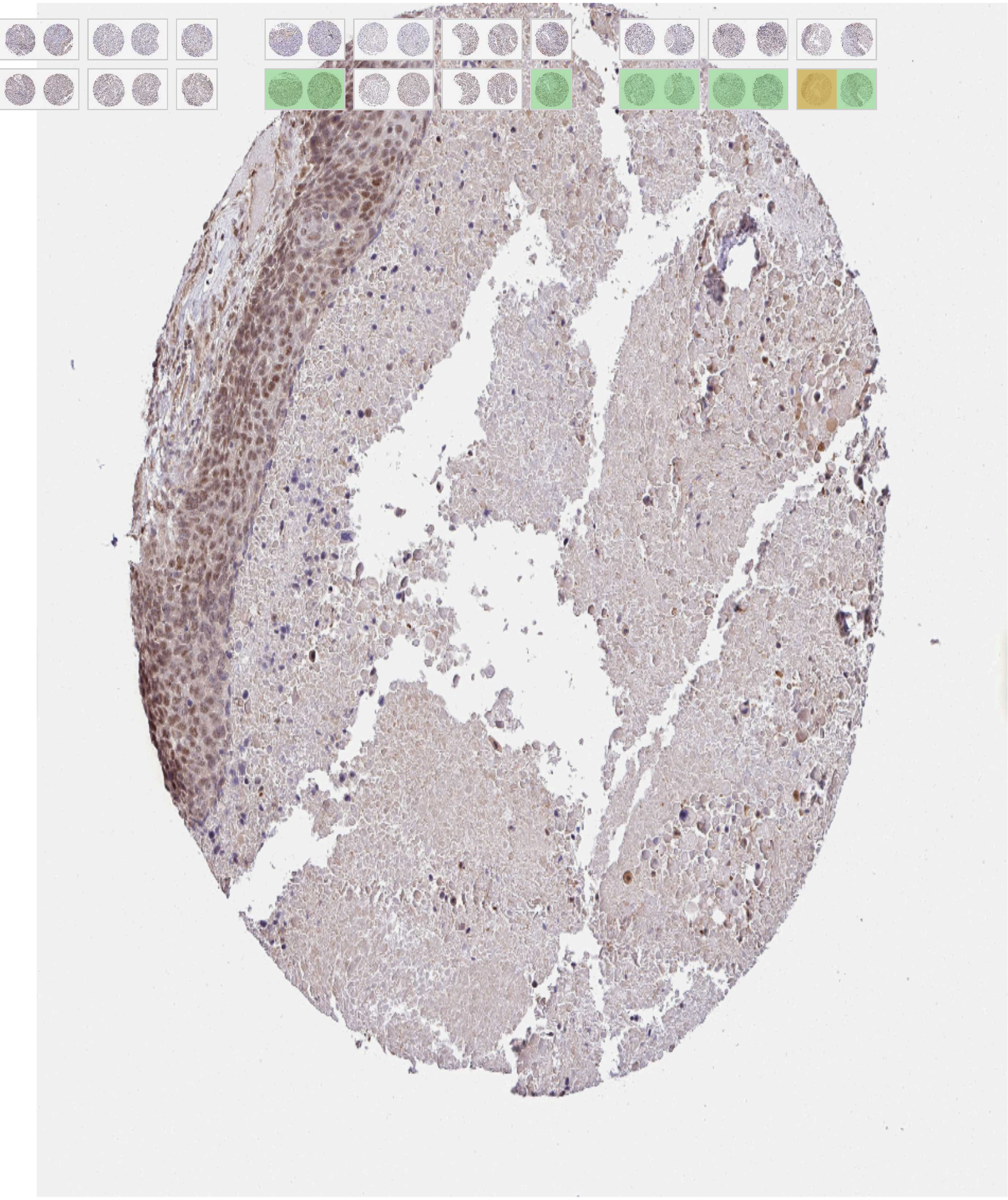

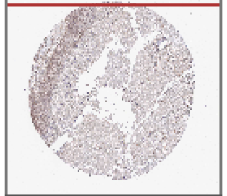

Lung cancer

HPA053229

Male, age 66
Lung (T-28000)
Lymph node (T-08000)
Squamous cell carcinoma, NOS (M-80703)
Normal tissue, NOS (M-00100)
Patient id: 3185

Tumor cells

Staining:	**Medium**
Intensity:	**Moderate**
Quantity:	**75%-25%**
Location:	**Nuclear**
