# Supplementary figures and images for: PLK1 overexpression as a dual-role biomarker and therapeutic vulnerability in pulmonary adenocarcinoma
Source: PeerJ. 2026 Jan 15;14:e20618. doi: 10.7717/peerj.20618 (PMC12812279; doi:10.7717/peerj.20618)

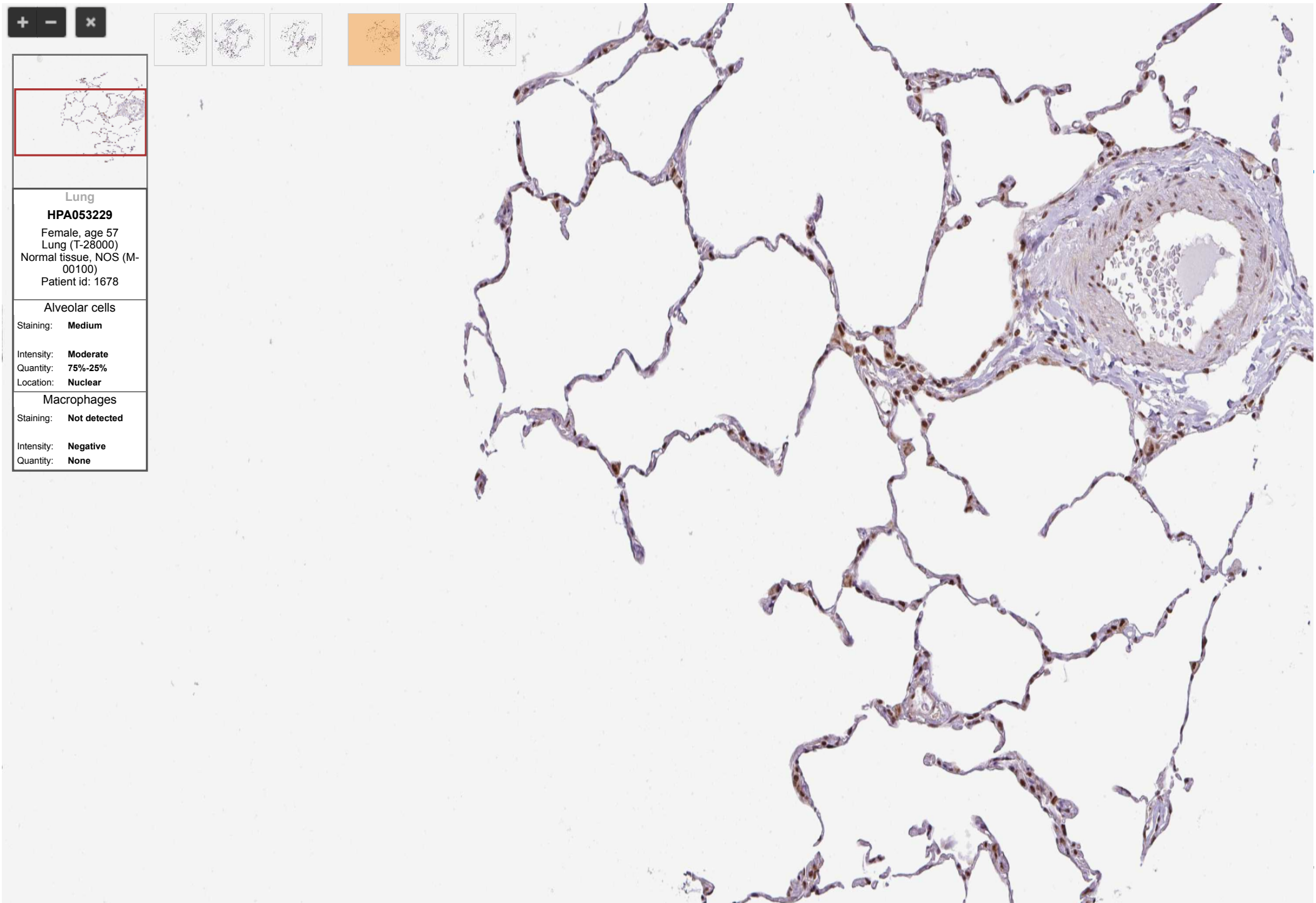

Supplement: Supplemental Information 2 [file peerj-14-20618-s002.tar › additional files/Figure 1F Tissue expression of PLK1 - Staining in lung - The Human Protein Atlas.pdf]

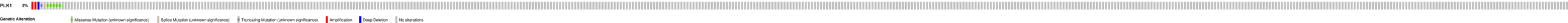

Supplement: Supplemental Information 2 [file peerj-14-20618-s002.tar › additional files/Figure 1G oncoprint.pdf]

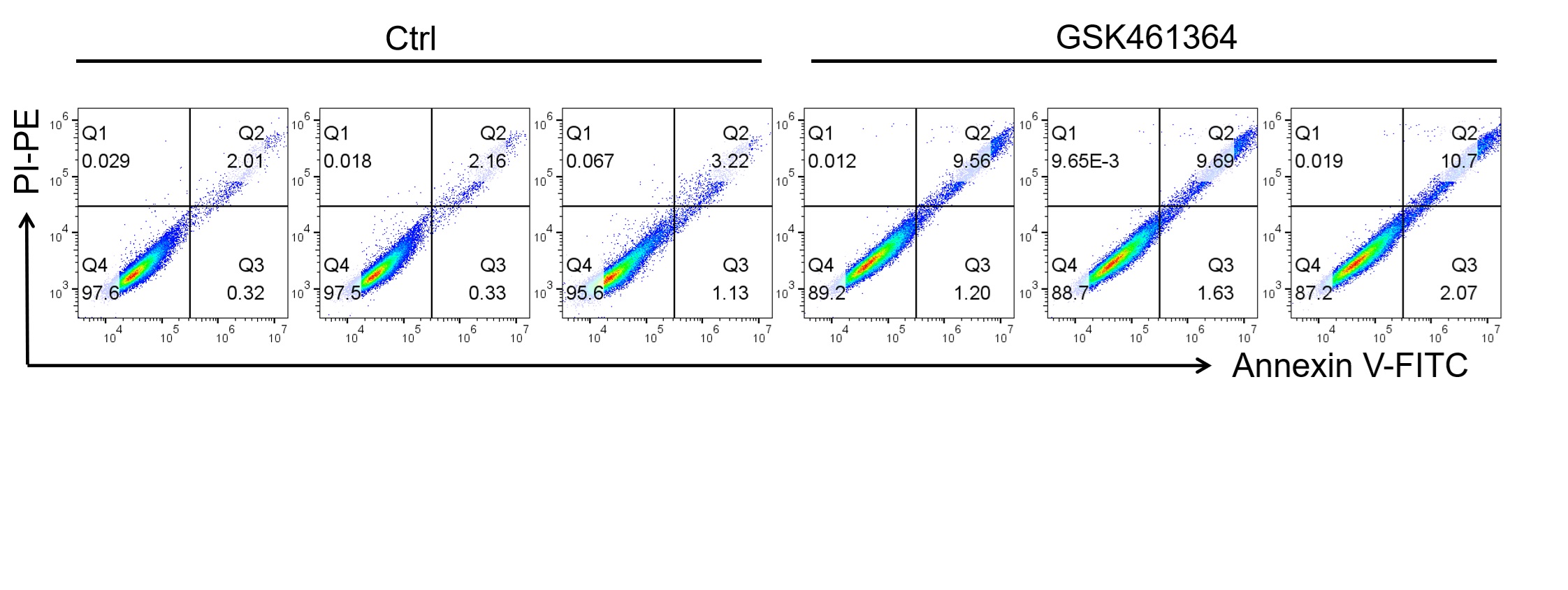

Supplement: Supplemental Information 2 [file peerj-14-20618-s002.tar › additional files/Figure 7D flow cytometry plots for apoptosis.jpg]

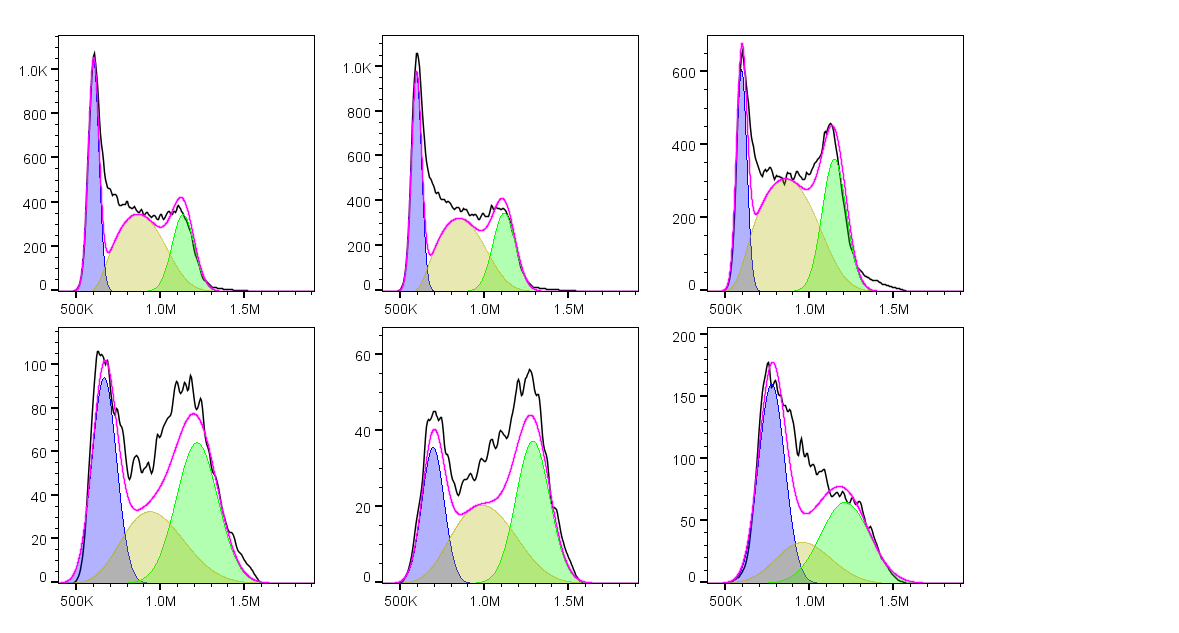

Supplement: Supplemental Information 2 [file peerj-14-20618-s002.tar › additional files/Figure 7F flow cytometry plots for cell cycle.png]
